# GSG2 facilitates the progression of human breast cancer through MDM2-mediated ubiquitination of E2F1

**DOI:** 10.1186/s12967-023-04358-2

**Published:** 2023-08-03

**Authors:** Yu Tang, Gaosai Dai, Yupeng Yang, Huantao Liu

**Affiliations:** 1 Day Ward, Cancer Hospital of China Medical University, Liaoning Cancer Hospital and Institute, No. 44 Xianheyan Road, Shenyang, 110042 China; 2 Department of Breast Surgery, Qilu Hospital of Shandong University, No. 107 Wenhuaxi Road, Jinan, 250012 Shandong China; 3 Department of Thyroid and Breast Surgery, Jinan Zhangqiu District Hospital of TCM, Xiushui Street 1463, Jinan, 250200 Shandong China

**Keywords:** BC, GSG2, Prognosis, Phenotype, E2F1, Ubiquitination

## Abstract

**Background:**

Breast cancer (BC) has posed a great threat to world health as the leading cause of cancer death among women. Previous evidence demonstrated that germ cell-specific gene 2 (GSG2) was involved in the regulation of multiple cancers. Thus, the clinical value, biological function and underlying mechanism of GSG2 in BC were investigated in this study.

**Methods:**

The expression of GSG2 in BC was revealed by immunohistochemistry (IHC), qPCR and western blotting. Secondly, the biological function of GSG2 in BC was evaluated by MTT assay, flow cytometry, Transwell assay and wound healing assay. Furthermore, the potential molecular mechanism of GSG2 regulating the progression of BC by co-immunoprecipitation (Co-IP) and protein stability detection.

**Results:**

Our data indicated that GSG2 was frequently overexpressed in BC. Moreover, there was a significant correlation between the GSG2 expression and the poor prognosis of BC patients. Functionally, GSG2 knockdown inhibited the malignant progression of BC characterized by reduced proliferation, enhanced apoptosis and attenuated tumor growth. Migration inhibition of GSG2 knockdown BC cells via epithelial-mesenchymal transition (EMT), such as downregulation of Vimentin and Snail. In addition, E2F transcription factor 1 (E2F1) was regarded as a target protein of GSG2. Downregulation of E2F1 attenuated the promoting role of GSG2 on BC cells. Mechanistically, knockdown of GSG2 accelerated the ubiquitination of E2F1 protein, which was mediated by E3 ubiquitin ligase MDM2.

**Conclusions:**

GSG2 facilitated the development and progression of BC through MDM2-mediated ubiquitination of E2F1, which may be a promising candidate target with potential therapeutic value.

**Supplementary Information:**

The online version contains supplementary material available at 10.1186/s12967-023-04358-2.

## Background

Breast cancer (BC) is often referred to as the “pink killer”, and its incidence ranks first among female malignant tumors [1]. In addition, BC is a heterogenous disease composed of three distinct subtypes (hormone receptor expression with different molecular characteristics and genetic profiles, including estrogen receptor (ER+) or progesterone receptor (PR+), human epidermal receptor 2 expression (HER2+), and triple negative BC (TNBC) (ER−, PR−, HER2− [2, 3]. The management of BC varies due to its different molecular characteristics [4]. Currently, the treatment strategy for BC includes surgery, chemotherapy, and radiotherapy combined with endocrine therapy [4]. In recent years, there are several types of targeted therapies used in BC treatment, including hormone therapy, HER2-targeted therapy, CDK4/6 inhibitors and PARP inhibitors [5]. Although these treatments have been effective, the complex mechanism of tumorigenesis and development for BC still hinders the treatment of the disease [6]. Therefore, in order to achieve more effective treatment management options, it is necessary to focus on the key factors of osteosarcoma progression to identify more targeted therapies for BC treatment.

Protein kinases exhibit roles in many cellular processes such as gene expression, membrane transport, metabolism, proliferation, apoptosis, movement [7]. Germ cell-specific gene 2 (GSG2), also termed haploid germ cell specific nuclear protein kinase (Haspin), is an atypical serine/threonine protein kinase [8], which is required for proliferation and differentiation in mammalian cells [9]. GSG2 is responsible for the phosphorylation of histones, particularly histone H3 at Thr3 (H3T3) during mitosis, which serves a role in chromosome segregation [10, 11]. Accumulating evidences demonstrate that GSG2 expression is abnormally elevated in a variety of cancers, such as pancreatic cancer [12], bladder cancer [13], gallbladder carcinoma [14] and BC [15].

Furthermore, GSG2 inhibition delays cell cycle progression through interphase in cancer cells, which has a great potential as an anticancer therapeutic target [16, 17]. Moreover, inhibition of GSG2 may overcome melanoma resistance, modulate the immune environment, and target the vulnerability of different cancer lineages [18]. Recently, Huang et al., demonstrated that GSG2 is involved in the development of ovarian cancer and has the potential to be a therapeutic target and prognostic indicator for ovarian cancer treatment [19]. Geng et al., reported that GSG2 knockdown significantly inhibited various malignant biological behaviors of esophageal cancer cells, such as inhibiting proliferation, reducing colony formation, promoting apoptosis, and blocking migration [20]. Li et al., revealed that GSG2 promoted tumor growth through regulating cell proliferation in hepatocellular carcinoma [21]. Till now, the clinical value and biological function of GSG2 in BC have not been clarified, which arouses our interest.

In the present study, the expression level of GSG2 in BC and its prognostic correlation were revealed. Secondly, the biological function of GSG2 in BC has been explored separately in vitro and in vivo. More importantly, the potential molecular mechanism of GSG2 regulating the progression of BC had been initially explored. Altogether, the effects of GSG2 in the development and progression of BC were investigated.

## Methods

### Clinical BC tissue collection

The tumor tissues (n = 128) and paired normal tissues (n = 12) of human survival BC patients were purchased from Xi’an Alina Biotechnology Co., Ltd (Xi’an, China), including pathological details, such as age, gender, post-operative tumor grade and stage. None of the BC patients received radiotherapy or chemotherapy prior to biopsy sampling. The experimental procedures were approved by the Ethics Committee of Qilu Hospital of Shandong University and informed consent of the BC patients.

### Immunohistochemistry (IHC) staining and analysis

Formalin-immobilized BC and paired normal tissues were dewaxed in xylene, rehydrated in ethanol solution, incubated with 3% hydrogen peroxide to block endogenous peroxidase and non-specific binding sites. Next, the tissues were incubated with the primary antibody (anti-GSG2,1:100, BIOSS, Cat. #bs-14356R), (anti-E2F1, 1:100, abcam, Cat. # ab179445) at 4 °C overnight and the secondary antibody HRP goat anti-rabbit IgG (1:200, Beyotime, A0208) at room temperature for 1 h. Afterwards, tissues were stained with 3,3-diaminobenzidine solution for 3 min and the nuclei were counterstained with hematoxylin. The staining intensity was scored according to the criteria described in the literature [22]. The result greater than or equal to median values of IHC was defined as high expression, otherwise low expression.

### Cell culture

Human mammary epithelial cell HBL-100, BC cell lines BT549, MCF-7 and MDA-MB-231 were cultured in Dulbecco’s modified eagle medium (DMEM) (Gibco, Cat. #41,965,062) supplemented with 20% fetal bovine serum (FBS) (Gibco, Cat. #10091-148) and maintained 5% CO_2_ at 37 °C.

### Lentiviral shRNA vector construction and cell transfection

Short hairpin RNAs (shRNA) target human GSG2 (shGSG2), E2F1 (shE2F1) and scrambled sequences (shCtrl) were digested using AGE I (5′-ACCGGT-3′, 10 U/µl, NEB) and EcoR I (5′-GAATTC-3′, 10 U/µl, NEB) and inserted into the lentiviral vector BRV-112 that labeled with green fluorescent protein (GFP) (Bioscienceres, Shanghai, China). Meanwhile, the amplified sequence of GSG2 (GSG2) was synthesized and inserted into BRV-112 for overexpression of GSG2. Subsequently, 5 × 10^6^ MCF-7 and MDA-MB-231 cells were cultured for 24 h and transfected with recombinant BRV-112 vector containing shGSG2, GSG2 + NC-shE2F1, shE2F1 + NC-GSG2, GSG2 + shE2F1 and NC(OE + KD) (empty vector, as negative control) (1 × 10^8^ TU/ml) at a MOI (multiplicity of infection) of 10, respectively using Lipofectamine® 3000 (Invitrogen) at 37℃. After cultured for 72 h, the expression of GFP was observed and evaluated under a fluorescence microscope (200× magnification, OLYMPUS). The sequences as follows: shGSG2-1, 5′-CCACAGGACAATGCTGAACTT-3′, shGSG2-2, 5′-AAGGAAACTGGTGGTGGGAAA-3′, shGSG2-3, 5′-AGGGATTGACTTAGAGCAAAT-3′; shE2F1-1, 5′- GGGCATCCAGCTCATTGCCAA-3′, shE2F1-2, 5′- CAGCTGGACCACCTGATGAAT-3′, shE2F1-3, 5′- GACCTCTTCGACTGTGACTTT-3′; shCtrl: 5′-TTCTCCGAACGTGTCACGT-3′. BRV-112, 5′-CCGGTTCTCCGAACGTGTCACGTTTCAAGAGAACGTGACACGTTCGGAGAATTTTTG-3′.

### RNA extraction and quantitative real-time-polymerase chain reaction (qPCR)

RNA was extracted from MCF-7 and MDA-MB-231 cells using Trizol [23]. The cDNA was reverse transcribed by the Maxima First Strand cDNA Synthesis Kit (Thermo Fisher Scientific). Quantification of mRNA expression levels of GSG2 and E2F1 was accomplished by SYBR Green master mix (Thermo Fisher Scientific) by ABI Prism 7500 sequence detection system (Applied Biosystems) with normalization to the expression of GAPDH. The primer sequences were listed in Additional file 1: Table S1.

### Western blotting (WB) and co-immunoprecipitation (Co-IP)

MCF-7 and MDA-MB-231 cells protein was purified with RIPA (Beyotime) and the concentration was determined by bicinchoninic acid (BCA) Protein Assay Kit (Beyotime). The 20 µg/well total protein was subjected to 10% sodium dodecyl sulfate polyacrylamide gel electrophoresis (SDS–PAGE), transferred to polyvinylidene difluoride (PVDF) membrane (Millipore), hybridized with corresponding primary antibody (Additional file 1: Table S2) overnight at 4 °C, incubated with secondary antibody labeled with horseradish peroxidase (HRP) at room temperature for 2 h. Finally, protein signal was visualized using chemiluminescence ECL kit (Thermo Fisher Scientific) and GAPDH as load control.

A target protein-specific anti-GSG2 or anti-MDM2 antibody (Additional file 1: Table S2) in conjunction with Protein A/G affinity beads (Santa Cruz Biotechnology) for 30–60 min at 4 °C. The bead-antibody complexes were suspended with protein lysate. The beads were washed 3 times with extraction buffer, and collected by centrifugation at 3000*g*. Subsequently, the immunoprecipitants were subjected to WB.

### Methyl thiazolyl tetrazolium (MTT) assay

MCF-7 and MDA-MB-231 cells (2 ml/well) were digested by trypsin, resuspended into cell suspension, and cultured at a density of 2000 cells/well for 24 h, 48 h, 72 h, 96 and 120 h. The OD 490 nm value was detected under Microplate Reader after adding 20 µl 5 mg/ml MTT and 100 µl DMSO in turn. Finally, growth curve was drawing and analyzed by T-test method.

### Flow cytometry analysis of cell apoptosis

MCF-7 and MDA-MB-231 cells were inoculated into 6-well plates (2 ml/well) for 5 days. The cells were digested by trypsin, resuspended into cell suspension, fixed with ethanol, stained with propidium iodide (PI) (Sigma, Cat. #P4170). Afterwards, the cells were successively washed and precipitated by PBS and 1 × binding buffer, stained with 5 µl annexin V-APC in the dark for 15 min and then stained with 5 µl PI again. The apoptosis rate was measured by flow cytometry and the results were analyzed by T-test.

### Human apoptosis antibody array assay

In this experiment, the apoptosis antibody array kit (Abcam, USA, Cat. #ab134001) was used to detect the expression of proteins related to human apoptosis signaling pathway. MCF-7 cells protein was diluted with the array diluent buffer kit to 0.5 mg/ml. The protein was transferred to antibody membrane, blocked with blocking buffer for 30 min at room temperature and incubated with HRP linked streptavidin overnight at 4 °C. Finally, protein signal was observed with ChemiDoc XRS chemiluminescence and imaging system. The gray value was quantitated using Quantity One software and normalized to the *α*–tubulin levels.

### Transwell assay

MCF-7 and MDA-MB-231 cells were digested by trypsin, resuspended into cell suspension (80,000 cells/well) and placed into Transwell inner chambers (24-well, 8-mm pore) (Corning) 100 µl. 500 µl DMEM medium containing 30% FBS was added in the outer chambers. After incubation 24 h at 37 °C, the migratory cells on the lower surface of Polycarbonate membrane were fixed with 4% precooled paraformaldehyde for 30 min and stained with 0.1% of crystal violet for 20 min at room temperature. Following washing with PBS, the cells in 5 fields were randomly selected under a fluorescence microscope (200× magnification, OLYMPUS), and the migration rate was calculated according to the number of migratory cells.

### Wound-healing assay

MCF-7 and MDA-MB-231 were seeded into 96-well plats. After cell growing for 24 h, the complete medium was changed into medium with lower serum concentration. Then, we scratched a wound across the cell layer using a 96-wounding replicator (Cat. #VP408FH, VP scientific). Cell debris were slightly rinsed with serum-free medium for 2–3 times. Images were captured at 0 and 24 h under a fluorescence microscope (50× magnification, OLYMPUS). Cell migration rate of each group were calculated based on the migration distance.

### In vivo xenograft model of mice

The experimental procedures performed on the mice were approved by the Ethics Committee of Qilu Hospital of Shandong University and accordance with Guide for Care and Use of Laboratory animals (NIH publication number 85-23, revised at 1996). The total of 20 BALB/c nude SPF mice (18 to 23 g, 4 weeks) (Lingchang biological, Shanghai) were kept in a sterile environment and fed normally. Lentivirus-transfected MDA-MB-231 cells were digested with trypsin and injected into the right forearm of mice with 500 µl (8 × 10^6^ cells/mouse) and divided into shCtrl (n = 10) and shGSG2 (n = 10) groups. Twelve days after cell injection, the tumor size was monitored once or twice a week and tumor volume was calculated as: π/6×L×W×W (L represented long diameter and W represented short diameter). Mice were anesthetized with 0.7% sodium pentobarbital (10 µl/g) and the tumor load was assessed by fluorescence imaging under the IVIS spectroscopic imaging system (emission wavelength of 510 nm). 49 days after the subcutaneous injection, the mice were sacrificed (the mice were grasped by the right hand and pulled back, the thumb and index finger of the left hand were pressed on the head of the mouse, and the spinal cord and brain were severed), the tumor was removed and weighed. Finally, IHC staining was used to detect the expression of KI67 (1:200, Abcam, Cat. #ab16667) in tumor tissues of each group.

### Microarray

RNA was purified from MDA-MB-231 cells and sequenced using Affymetrix human Gene Microarray Prime View (Affymetrix Scanner 3000 scan). The differentially expressed genes (DEGs) was recognized with criterion of |Fold Change| ≥ 1.8 and false discovery rate (FDR) ≤ 0.05 and presented as hierarchical clustering. Significant enrichment of DEGs in disease and function was investigated based on Ingenuity Pathway Analysis (IPA).

### Bioinformatics analysis

The human E3 ubiquitin ligase-substrate interaction network was explored on UbiBrowser (http://ubibrowser.ncpsb.org). In order to reveal the impact of GSG2 knockdown on downstream genes and signaling pathways, we used the database for Annotation Visualization and Integrated Discovery (DAVID, https://david.ncifcrf.gov/) to analyze the Kyoto Encyclopedia of Genes and Genomes (KEGG).

### Statistical analysis

All experiments were accomplished in triplicate and data were shown as mean ± SD. Statistical analyses and graphs were performed by GraphPad Prism 7.0 (Graphpad Software) and P value < 0.05 as statistically significant. The significance difference between two groups were determined using the two-tailed Student’s T test or One-way ANOVA analysis.

## Results

### Correlation analysis between GSG2 expression and BC

**Fig. 1:**
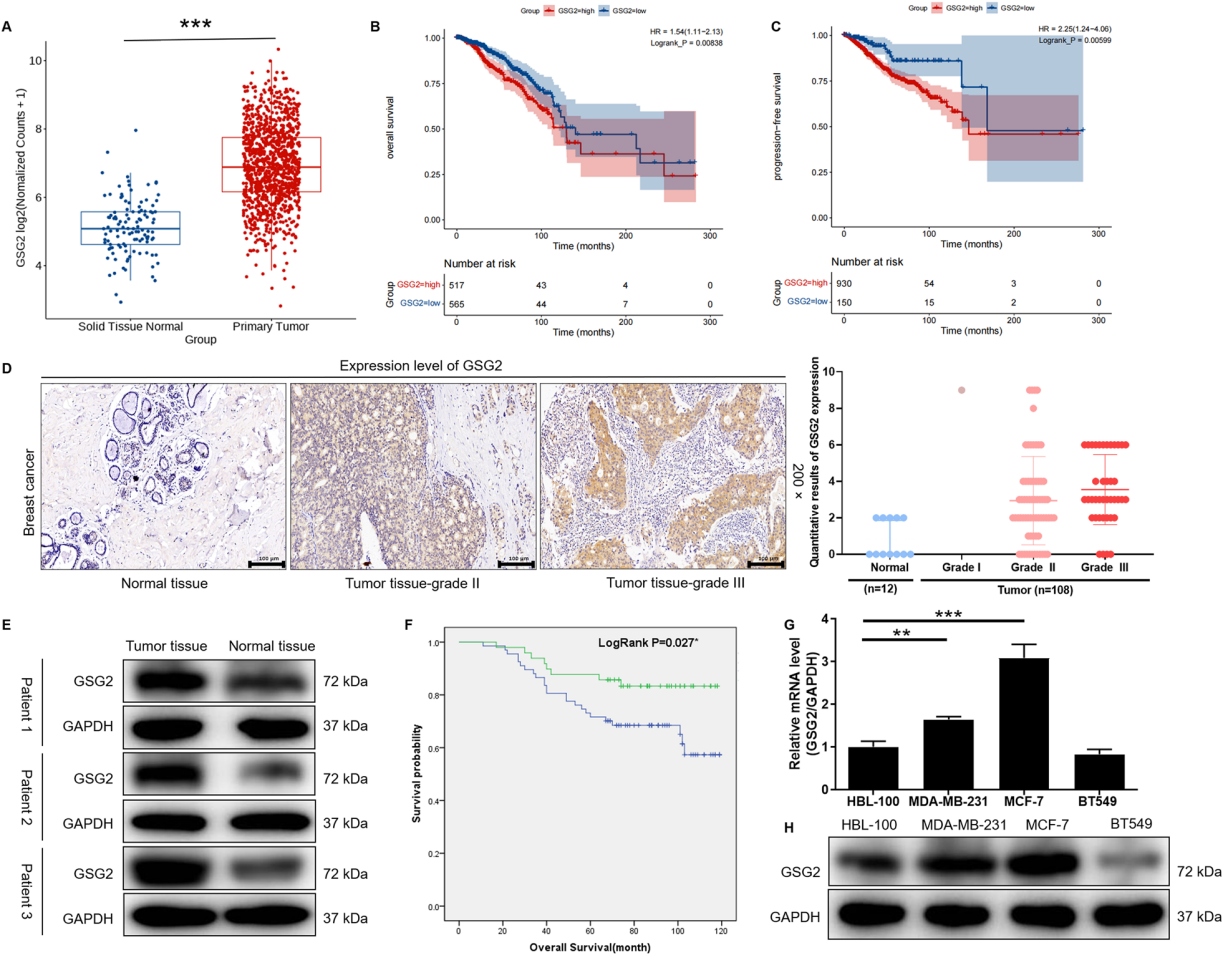
GSG2 expression was significantly elevated in human BC and possessed clinical value in predicting poor prognosis. **A** According to the TCGA database, the expression of GSG2 in BC tumor tissue and normal tissue was revealed. **B**, **C** The samples were divided into high GSG2 expression group and low GSG2 expression group, and the difference in overall survival or progression-free survival between the two groups was analyzed by log rank test. **D** The expression level of GSG2 in tumor tissues and normal tissues of BC patients was determined by IHC staining and representative images were shown. Magnification was 200. Quantitative results of GSG2 expression in normal and various breast tumor grades. **E** WB revealed the protein expression of GSG2 in normal and tumor tissues of BC patients. **F** The clinical relevance between GSG2 and overall survival of BC patients was analyzed by Kaplan-Meier method. *P = 0.027. **G**, **H** The mRNA and protein level of GSG2 in BC cell lines (McF-7, MDA-MB-231 and BT549) and normal mammary epithelial cell line HCL-100 was evaluated using qPCR. Data was represented as mean ± SD. **p < 0.01, ***p < 0.001

According to the analysis of BC and normal tissue samples in the TCGA database, the expression level of GSG2 in BC was significantly higher than that in normal tissue (P < 0.001; Fig. 1A). Afterward, the samples were divided into high GSG2 expression group and low GSG2 expression group, and the difference in overall survival between the two groups was analyzed by log rank test. The results showed that the overall survival time of high GSG2 expression group was significantly shorter than that of low expression group (P < 0.001; Fig. 1B). Similarly, the progression-free survival time of high GSG2 expression group was significantly shorter than that of low expression group (P < 0.001; Fig. 1C). Based on the TCGA database information, Cox multivariate regression analysis was performed to evaluate whether GSG2 expression could be used as an independent prognostic factor by comparing the expression level of GSG2 and the clinical characteristics of BC patients. The data indicated that GSG2 was not significantly associated with prognosis (P = 0.057; Additional file 1: Table S3).

In order to determine the clinical significance of GSG2 in BC, we analyzed the GSG2 levels in 128 BC patients. Representative images of IHC staining for BC patients’ tissues were shown in Fig. 1D, GSG2 was higher expressed in tumor tissue samples than that in the corresponding normal tissues. According to the IHC score, greater than or equal to 5 was defined as high expression, while less than 5 was defined as low expression. High expression of GSG2 was observed in 73 of 128 tumor tissue (57%) and in 0 of 12 normal tissues (P < 0.001) (Table 1). Consistently, the results of WB confirmed the up-regulation of GSG2 expression in BC tumor tissue (Fig. 1E). Based on the clinicopathological information of BC patients, the relationship between GSG2 expression and clinicopathologic features were investigated, such as age, grade, T infiltrate, N stage, The American Joint Committee on Cancer (AJCC) stage and tumor size. We found that GSG2 expression was significantly associated with grade (P = 0.033) (Table 2). Moreover, clinical data showed that the GSG2-high and GSG2-low cases indicating a significant difference in overall survival (P = 0.027) (Fig. 1F). Therefore, we demonstrated at the clinical level that GSG2 expression was upregulated in BC and was significantly associated with overall survival.

**Table 1:** Expression patterns in breast cancer tissues and para-carcinoma tissues revealed in immunohistochemistry analysis

GSG2 expression	Tumor tissue		Normal tissue		P value
	Cases	Percentage	Cases	Percentage	
Low	55	43%	12	100%	< 0.001
High	73	57%	0	–	

**Table 2:** Relationship between GSG2 expression and tumor characteristics in patients with breast cancer

Features	No. of patients	GSG2 expression		p value
		Low	High	
All patients	116	49	67	
Age (years)				0.852
≤ 58	58	24	34	
> 58	58	25	33	
Grade				0.033
I	1	0	1	
II	67	34	33	
III	40	11	29	
T Infiltrate				0.968
T1	32	14	18	
T2	68	28	40	
T3	11	4	7	
T4	2	2	0	
N stage				0.696
N0	61	28	33	
N1	30	11	19	
N2	17	6	11	
N3	6	4	2	
AJCC stage				0.695
1	22	8	14	
2	62	28	34	
3	28	12	16	
Tumor size				0.835
≤ 3 cm	67	29	38	
> 3 cm	46	19	27	

### Knockdown of GSG2 inhibited proliferation, promoted apoptosis and impeded migration of BC cells

**Fig. 2:**
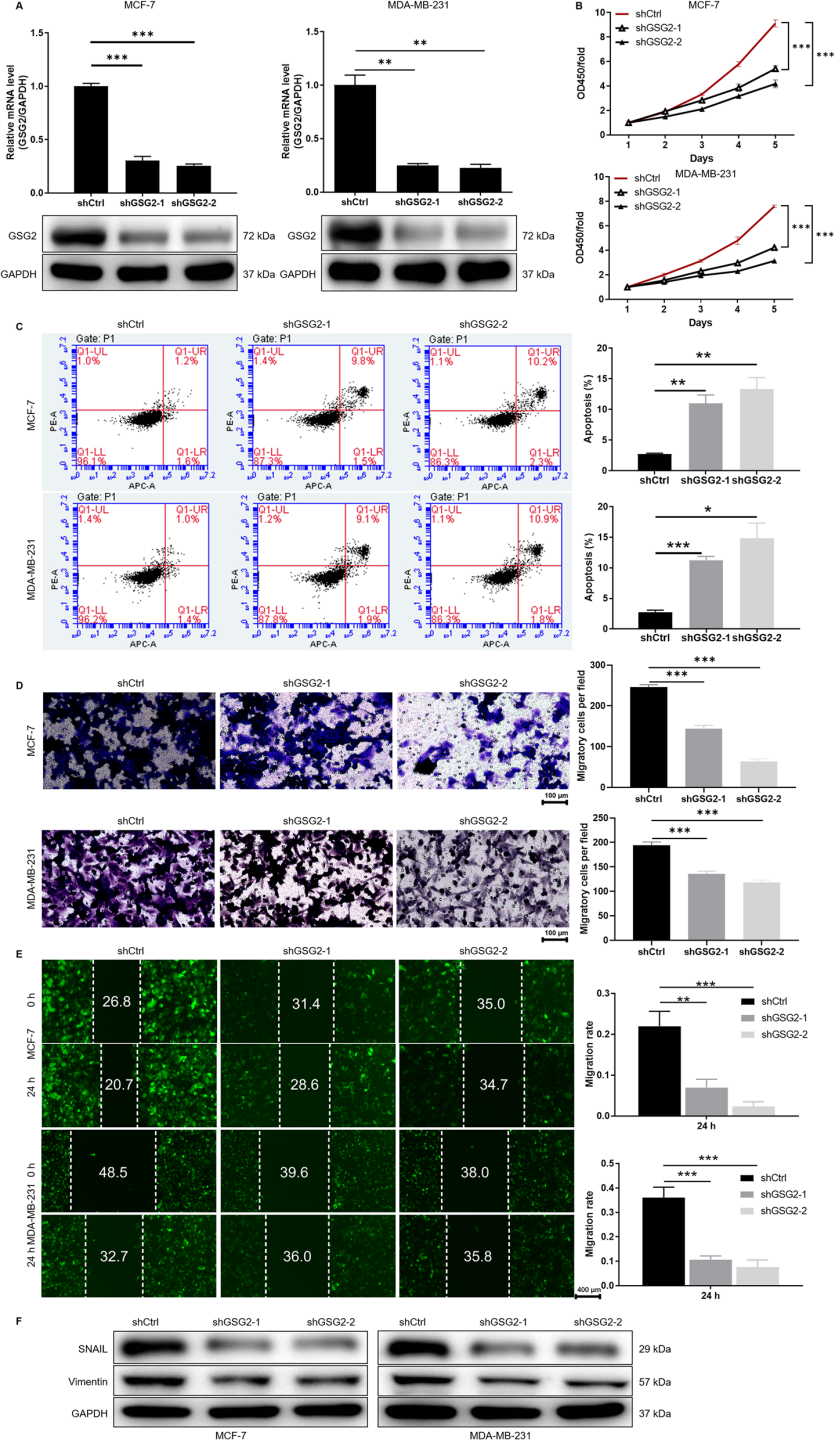
Knockdown of GSG2 inhibited proliferation, arrested cell cycle, promoted apoptosis and impeded migration of BC cells. **A** The specificity and validity of the lentivirus-mediated shRNA knockdown of GSG2 expression was verified by qPCR and WB. **B** The proliferation of MCF-7 and MDA-MB-231 cells after knockdown of GSG2 (shGSG2-1 and shGSG2-2) was measured using MTT assay. **C** Cell apoptosis of MCF-7 and MDA-MB-231 cells after knockdown of GSG2 (shGSG2-1 and shGSG2-2) was analyzed by flow cytometry. **D**, **E** The migration of MCF-7 and MDA-MB-231 cells after knockdown of GSG2 was measured using **D** Transwell assay and **E** wound-healing assay. **F** The protein expression of Vimentin and Snail of MDA-MB-231 cells after knockdown of GSG2 was measured by WB. shGSG2-1 and shGSG2-2 indicated GSG2 knockdown in MCF-7 and MDA-MB-231 cells; shCtrl indicated MCF-7 and MDA-MB-231 cells transfected with an empty vector. The presented results were representative of experiments repeated at least three times. Data was represented as mean ± SD. *P < 0.05, **P < 0.01, ***P < 0.001

Next, we further revealed the difference in the expression of GSG2 in BC cells and normal mammary epithelial cells at the cellular level. As illustrated in Fig. 1G, the mRNA level of GSG2 in BC cell line McF-7 and MDA-MB-231 was significantly higher than that in normal mammary epithelial cell line HCL-100 (P < 0.01). As expected, the protein expression of GSG2 was more abundant in BC cells than in normal breast epithelial cells (Fig. 1H). Subsequently, two shRNA sequences targeting GSG2 (shGSG2-1 and shGSG2-2) were transfected into breast cancer cell lines MCF-7 and MDA-MB-231, respectively (P < 0.01) (Additional file 1: Fig. S1A). The qPCR and WB results showed that GSG2 expression in MCF-7 and MDA-MB-231 cells was significantly downregulated in shGSG2-1 and shGSG2-1 groups compared with shCtrl (P < 0.01) (Fig. 2A). Thus, we examined cell phenotypes in MCF-7 and MDA-MB-231 cells after the interference of dual shGSG2-1 and shGSG2-2 targets.

Based to the results of MTT assay, we found that MCF-7 and MDA-MB-231 cells in shGSG2-1 and shGSG2-1 groups exhibited slower proliferation rate than that in shCtrl group (P < 0.001) (Fig. 2B). Furthermore, we examined the cell apoptosis in response to knockdown of GSG2. The apoptotic rate of MCF-7 and MDA-MB-231 increased significantly after reducing the expression of GSG2 (P < 0.05) (Fig. 2C). Meanwhile, GSG2 knockdown inhibits the expression of anti-apoptotic proteins Bcl-2, Bcl-W, IGF-I, IGF-II, IGF-1SR, Livin, Survivin, STNF-R1 and TNF-β in BC cells (P < 0.05) (Additional file 1: Fig. S1B).

Additionally, the effects of GSG2 knockdown on the migration of BC cells were evaluated by Transwell and wound-healing experiments. In Fig. 2D, the number of migratory MCF-7 and MDA-MB-231 cells in shGSG2-1 and shGSG2-1 groups was significantly less than that in shCtrl group (P < 0.001). The monitoring results in the 0–24 h period showed that the wound healing ability of GSG2 knockdown BC cells was weakened (P < 0.01) (Fig. 2E). As a result, our data revealed that GSG2 knockdown weakened the migration of MCF-7 and MDA-MB-231 cells. In addition, epithelial-mesenchymal transition (EMT) is a developmental process that promotes migration and metastasis in various types of tumors. During EMT, Vimentin, and Snail are the most commonly detected epithelial and mesenchymal markers, respectively [24, 25]. In this study, we found that GSG2 knockdown in MCF-7 and MDA-MB-231 cells resulted in downregulation of Vimentin and Snail (Fig. 2F). Therefore, GSG2 knockdown in MCF-7 and MDA-MB-231 cells could regulate cell biological behaviors, such as inhibiting proliferation and migration, enhancing apoptosis. In addition, inhibition of migration may be mediated by EMT in BC cells.

### Knockdown of GSG2 suppressed tumor growth in the mouse xenograft model

**Fig. 3:**
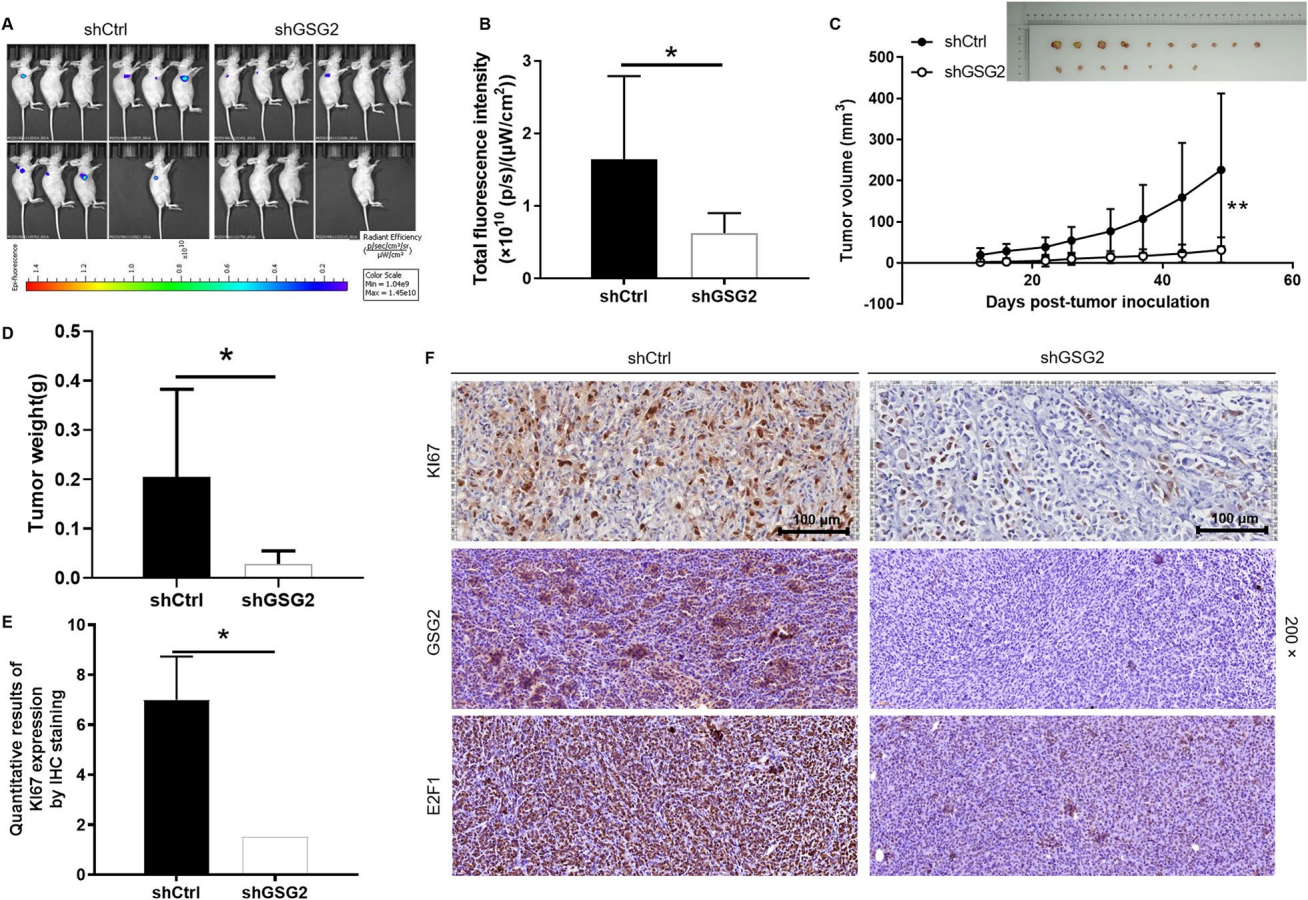
Knockdown of GSG2 attenuated tumor formation of BC in vivo. **A** The total fluorescence intensity was scanned using in vivo imaging system and used as a representation of tumor burden in mice. **B** Post injection of MDA-MB-231 cells for 12 days, the tumor volume in mice was measured. **C** Mice were sacrificed at day 49 post injection, and the tumor weight was measured. **D** The expression of KI67 in mouse tumor tissues was detected by IHC staining. Magnification was 200. **E** Quantitative results of KI67 expression in mice tumor. **F** IHC staining was performed in mouse tumor tissues to clarify the expressions of KI67, GSG2 and E2F1. Magnification was 200. The presented results were representative of experiments repeated at least three times. Data was represented as mean ± SD. *P < 0.05, **P < 0.01

The effects of GSG2 knockdown on BC was further elucidated in the mice xenograft model. Lentivirus-transfected MDA-MB-231 cells were injected into mice and the tumorigenesis rate was 100% and 70% in shCtrl and shGSG2 groups, respectively. Tumor load of mice was observed by fluorescence intensity, founding that the intensity of shGSG2 group was significantly weaker than that of the control group (P < 0.05) (Fig. 3A and B). After the mice were subcutaneously injected with MDA-MB-231 cells for 49 days, the growth of tumors in the shGSG2 group was almost stagnant, while those in the shCtrl group grew faster (P < 0.01) (Fig. 3C). Tumors in each group were removed and weighed, which demonstrated that the shGSG2 group was significantly lighter than the shCtrl group (P < 0.05) (Fig. 3D). Consistently, the results of IHC showed that the expression level of KI67 in shGSG2 group was significantly lower than that in control group (P < 0.05) (Fig. 3E). Moreover, IHC staining images of mouse tumor tissue confirmed that GSG2 and E2F1 expression was reduced in the GSG2 knockout group compared with the control group (Fig. 3F). As a results, we demonstrated that downregulation of GSG2 could inhibit tumor growth in vivo.

### E2F1 was the downstream target of GSG2 regulating BC

**Fig. 4:**
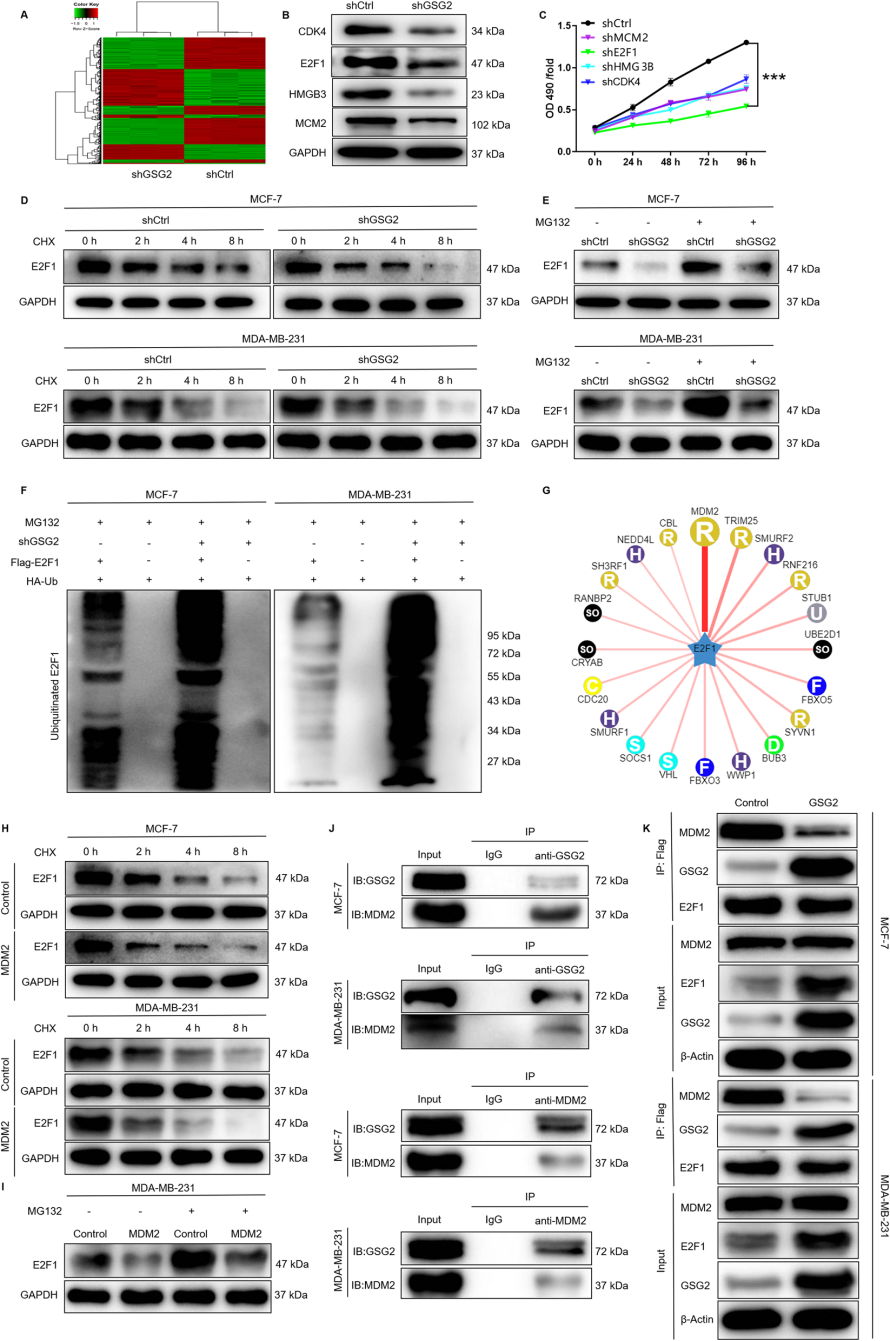
Knockdown of GSG2 results in the alteration of downstream proteins. **A** The DEGs between shGSG2 and shCtrl groups of MDA-MB-231 cells was identified. In the heat map of cluster analysis, each column represents a sample and each row represents a differential gene. The red indicates that the gene expression is upregulated, the green indicates that the gene expression is downregulated, the black indicates that the gene expression is not significantly changed, and the gray indicates that the signal strength of the gene is not detected. **B** The most significant DEGs were further screened by western blot analysis. **C** The effect of CDK4, E2F1, HMGB3, and MCM2 knockdown on the viability of MDA-MB-231 cells. **D** The protein stability of E2F1 in MCF-7 and MDA-MB-231 cells after GSG2 knockdown was examined. **E** MCF-7 and MDA-MB-231 cells with GSG2 knockdown were treated with MG-132, and the stability of E2F1 protein was tested. **F** The lysates of MCF-7 and MDA-MB-231 cells after GSG2 knockdown were immunoprecipitated and WB was performed to examine the ubiquitination of E2F1. **G** Several E3 ubiquitin ligases affecting the stability of E2F1 protein were predicted by UbiBrowser analysis. **H** The protein stability of E2F1 in MCF-7 and MDA-MB-231 cells after MDM2 overexpression were examined. **I** MDA-MB-231 cells with MDM2 overexpression were treated with MG-132, and the stability of E2F1 protein was tested. **J** The protein interaction between GSG2 and MDM2 was verified by forward and reverse CO-IP, respectively. **K** Immunoprecipitation was used to detect the binding of MDM2 and E2F1 in MCF-7 and MDA-MB-231 cells that stably overexpressed GSG2. The presented results were representative of experiments repeated at least three times. Data was represented as mean ± SD. ***P < 0.01

The molecular mechanism of GSG2 regulating BC cells was initially explored through human gene microarray. The results of microarray indicated that knockdown of GSG2 resulted in upregulation of 433 genes and downregulation of 541 genes (Fig. 4A, Additional file 2: Table S4). Furthermore, Gene Set Enrichment Analysis (GSEA) was carried out based on shGSG2 vs. shCtrl expression profile data. Ranked by significance P value, the top 10 gene sets that were most significantly concentrated in the classical pathways of KEGG, BIOCARTA, PID, REACTOME and WIKIPATHWAYS databases are shown in the Additional file 1: Fig. S2A, S2B. The significant enrichment of DEGs in diseases and functions showed that cancer, cell death and survival, cell cycle, cellular growth and proliferation were significantly inhibited (Additional file 1: Fig. S2C). Moreover, phosphatidylinositol 3-kinase (PI3K)/protein kinase B (AKT) signaling pathway is involved in proliferation, survival, migration, apoptosis and other biological processes of BC [26]. Cyclin D1 (CCND1)/cyclin-dependent kinase 1 (CDK1) is essential for maintaining the orderly progression of the cell cycle [27]. The present study revealed that knockdown of GSG2 leaded to alterations in the core components of the signaling pathways, such as decreased phosphorylation of AKT, downregulation of PIK3CA, CCND1 and CDK1 (Additional file 1: Fig. S2D, S2E). Subsequently, the most significant DEGs were further screened by qPCR and western blot analysis, indicating that the expression of CDK4, E2F1, HMGB3, and MCM2 in the shGSG2 group was downregulated compared to the shCtrl group (Fig. 4B). Moreover, lentivirus was used to deliver shRNAs into MDA-MB-231 cells to knockdown CDK4, E2F1, HMGB3, and MCM2, respectively. Compared with other groups, MDA-MB-231 with E2F1 knockdown showed the strongest inhibitory effect on proliferation (P < 0.001) (Fig. 4C). The above results suggested that E2F1 may be relevant to the progression of BC. Thus, the mechanism by which GSG2 regulated BC and its relationship with E2F1 had attracted our attention.

### GSG2 regulated the ubiquitination of E2F1 through the E3 ubiquitin ligase MDM2

In order to reveal the mechanism of GSG2 regulating E2F1 protein expression, we conducted the following exploration. MCF-7 and MDA-MB-231 cells transfected with lentiviral shCtrl and shGSG2 were treated with CHX (protein synthesis inhibitor, 0.2 mg/mL) for 0–8 h and the protein stability of E2F1 was tested, indicating that GSG2 knockdown accelerated the degradation of E2F1 protein (Fig. 4D). Notably, the effects of GSG2 knockdown on E2F1 protein stability could be partially eliminated by the treatment of MG-132, an inhibitor of proteasome (Fig. 4E), suggesting that GSG2 may mediate E2F1 protein stability by the ubiquitin-proteasome system (UPS). Subsequently, we evaluated the regulation of GSG2 on ubiquitination of E2F1 and the results indicated that GSG2 downregulation distinctly promoted E2F1 ubiquitination (Fig. 4F). Therefore, the above data suggested that GSG2 knockdown may promote ubiquitination degradation of E2F1 through UPS.

Considering that ubiquitination is a multi-step reaction process involving the participation of multiple ubiquitin ligases [28]. According to UbiBrowser analysis results, we predicted that the protein stability of E2F1 was most significantly affected by E3 ubiquitin ligase MDM2 (Fig. 4G). In addition, we further demonstrated that MCF-7 and MDA-MB-231 cells with overexpression of MDM2 induced a decrease of E2F1 protein stability (Fig. 4H). As expected, the effects of MDM2 overexpression on E2F1 protein stability could be partially eliminated by the treatment of MG-132 (Fig. 4I). Moreover, the protein interaction between GSG2 and MDM2 was verified by forward and reverse CO-IP, respectively (Fig. 4J). The results suggested that GSG2 regulated the ubiquitination of E2F1 through the E3 ubiquitin ligase MDM2. Additionally, immunoprecipitation was used to detect the binding of MDM2 and E2F1 in MCF-7 and MDA-MB-231 cells that stably overexpressed GSG2. As showed in Fig. 4K, GSG2 overexpression weakened the binding affinity between MDM2 and E2F1. Taken together, GSG2 interacted with MDM2 and prevented E2F1 degradation. Of note, the CO-IP was performed in MCF-7 and MDA-MB-231 cells treated with or without GSG2 kinase (CHR-6494). The results showed that GSG2 kinase treatment did not affect the interaction of GSG2-MDM2 (Additional file 1: Fig. S3A).

### Knockdown of E2F1 alleviated the promoting role of GSG2 overexpression in BC cells

**Fig. 5:**
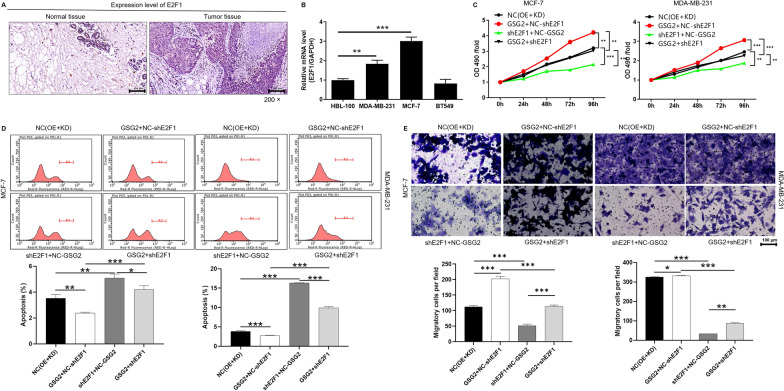
Knockdown of E2F1 alleviated the promoting role of GSG2 overexpression in BC cells. **A** E2F1 was highly expressed in tumor tissues compared with adjacent normal tissues in BC patients. Magnification was 200. **B** The mRNA level of E2F1 in BC cell lines (McF-7, MDA-MB-231 and BT549) and normal mammary epithelial cell line HCL-100 was evaluated using qPCR. (C-E) Detection of alteration in proliferation (**C**), apoptosis (**D**) and migration (**E**) in MDA-MB-231 cells. NC(KD + OE) indicated MDA-MB-231 cells transfected with empty vector, as negative control. GSG2 + NC-shE2F1 indicated GSG2 overexpression in MDA-MB-231 cells; shE2F1 + NC-GSG2 indicated E2F1 knockdown in MDA-MB-231 cells; GSG2 + shE2F1 indicated simultaneously upregulated GSG2 and downregulated E2F1 in MDA-MB-231 cells. The presented results were representative of experiments repeated at least three times. Data was represented as mean ± SD. *P < 0.05, **P < 0.01, ***P < 0.001

Additionally, the high expression of E2F1 in BC tissues and cell lines suggested that E2F1 may play a key role (Fig. 5A and B). Thus, the loss/gain-of function assays were conducted to further determine the effects of GSG2 and E2F1 in BC. As illustrated in the Fig. S3B, the shE2F1-2 sequences with the highest knockdown efficiency, which were used to construct E2F1 knockdown in MCF-7 and MDA-MB-231 cells. Notably, NC(OE + KD) was MDA-MB-231 cells transfected with empty vector, as negative control. GSG2 + NC-shE2F1 was the cells that overexpressed GSG2. shE2F1 + NC-GSG2 was the cells that downregulated E2F1. GSG2 + shE2F1 was MDA-MB-231 cells simultaneously overexpressing GSG2 and downregulating E2F1 (Additional file 1: Fig. S3C-S3D). Compared with negative control, BC cells with overexpression of GSG2 (GSG2 + NC-shE2F1) led to a promotion in proliferation and migration, while an inhibition in apoptosis (Fig. 5C and E). On the contrary, E2F1-knocked-down BC cells showed a significant inhibition of proliferation and migration, promotion of apoptosis (Fig. 5C and E). Interestingly, the malignant behaviors of MCF-7 and MDA-MB-231 cells in GSG2 + shE2F1 group was significantly suppressed compared with GSG2 + NC-shE2F1 (Fig. 5C and E). Collectively, the results showed that knocking down E2F1 can reduce the promotion of GSG2 overexpression on BC cells.

### Exploration of the effect of GSG2 and E2F1 on downstream signaling pathways

**Fig. 6:**
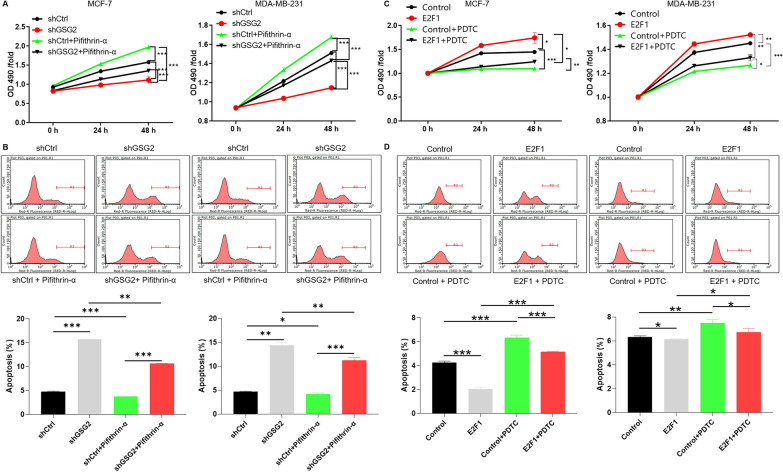
Exploration of the effect of GSG2 and E2F1 on downstream signaling pathways. **A**, **B** MCF-7 and MDA-MB-231 cells with GSG2 knockdown were treated with Pifithrin-α (p53 inhibitor, 50 µM) to elucidate the role of p53 in BC. **C**, **D** E2F1 overexpression BC cells were treated with PTDC (NF-κB inhibitor) to investigate the association with E2F1. The presented results were representative of experiments repeated at least three times. Data was represented as mean ± SD. *P < 0.05, **P < 0.01, ***P < 0.001

In addition, the results of KEGG indicated that GSG2 knockdown resulted in significant enrichment of p53 signaling pathway (Additional file 1: Fig. S3E). MCF-7 and MDA-MB-231 cells with GSG2 knockdown were treated with Pifithrin-α (p53 inhibitor, 50 µM) to elucidate the role of p53 in BC. Addition of Pifithrin-α could partially reverse the inhibitory effect of GSG2 knockdown on BC cells proliferation (Fig. 6A). Meanwhile, After GSG2 knockdown BC cells were treated with Pifithrin-α, their apoptotic rate slowed down (Fig. 6B). Accordingly, p53 may be involved in the process of GSG2 promoting BC cell progression.

Previous study reported that p53 suppressed E2F1 expression upon DNA damage by forming p53-E2F1-DNA complex [29]. Moreover, the transcriptional activity of E2F1 is regulated by NF-κB [30, 31]. Here, E2F1 overexpression BC cells were treated with NF-κB inhibitor to investigate the association with E2F1. Interestingly, MCF-7 and MDA-MB-231 cells with E2F1 overexpression showed proliferation promotion and apoptosis inhibition, and PDTC (NF-κB inhibitor) treatment partially alleviated these effects (Fig. 6C, D). Therefore, E2F1 may promote BC progression through NF-κB.

## Discussion

A significant breakthrough in this study is the identification of the promoting effect of GSG2 in human BC. It is well known that the BC is a complex, multifactorial process of heterogeneous disease associated with a variety of genetic abnormalities [4]. Previously, Zheng et al., revealed that GSG2 as a potential target for BC therapy based on the bioinformatics analysis results [32]. Consistently, we found that GSG2 was frequently upregulated in BC. In addition, there was a significant correlation between the GSG2 expression and the poor prognosis of BC patients. Therefore, GSG2 possessed great potential as a marker for early diagnosis and prognosis of BC.

More importantly, the malignant behaviors of GSG2 knockdown BC cells were significantly suppressed. In fact, GSG2 knockdown by shRNA specifically enhanced the apoptotic sensitivity of BC, while GSG2 overexpression resulted in reduced apoptotic capacity. Previous study clarified that apoptosis is the most important form of cell death and involves a series of cellular signals [13]. Our study indicated that GSG2 knockdown led to inhibition of Bcl-2, Bcl-W, IGF-I, IGF-II, IGF-1SR, Livin, Survivin, STNF-R1, and TNF-β of BC cells. On this basis, the alterations of key apoptotic factors after GSG2 knockdown were consistent with the observed apoptotic phenotype. Nonetheless, more exploration was required to better resolve how the molecular signaling pathways of apoptosis were regulated by GSG2 in BC.

On the other hand, knockdown of GSG2 impeded migration of MCF-7 and MDA-MB-231 cells. Metastasis is the leading cause of death in BC patients [33]. Epithelial-mesenchymal transformation (EMT) is crucial in promoting the invasion and migration of tumor cells during the development of cancer [25]. The process of EMT is regulated by a complex signal pathway and transcription factor network, which is characterized by the upregulation of Vimentin and Snail [34, 35]. In this study, WB analysis confirmed that GSG2 knockdown in BC cells triggered decreases of Vimentin and Snail. These results suggested that GSG2 knockdown may inhibit the migration of MCF-7 and MDA-MB-231 cells through EMT.

In addition, we found that the knock down of GSG2 resulted in a significant downregulation of E2F transcription factor 1 (E2F1) in BC. Moreover, GSG2 knockdown distinctly promoted E2F1 ubiquitination. A large number of studies have revealed and demonstrated that targeted ubiquitination has great therapeutic potential in a variety of cancers [36–39]. As we all know, ubiquitination is a multi-step reaction process involving the participation of multiple ubiquitin ligases [28]. According to UbiBrowser analysis results, we identified that the protein stability of E2F1 was most significantly affected by E3 ubiquitin ligase mouse double minute 2 (MDM2). Previous study reported that MDM2 stabilized E2F1 protein through the E2F1 ubiquitination pathway [40]. Here, we demonstrated that GSG2 regulated the ubiquitination of E2F1 through MDM2.

Furthermore, the results of KEGG indicated that GSG2 knockdown resulted in significant enrichment of p53 signaling pathway. We found that p53 may be involved in the process of GSG2 promoting BC cell progression. Previous study reported that p53 suppressed E2F1 expression upon DNA damage by forming p53-E2F1-DNA complex [29]. On the other hand, E2F1 possessed a crucial role in mediating multiple cancer hallmark capabilities that regulate metabolism, survival, cell cycle, apoptosis, and metastasis [41, 42]. Besides, E2F1 could be a potential therapeutic target in various human cancers, such as the patients with gastric cancer, ovarian cancer [43, 44]. Our study found that knockdown of E2F1 could alleviate the promoting role of GSG2 overexpression in BC cells. In addition, previous study reported that the transcriptional activity of E2F1 is regulated by NF-κB [30, 31]. The present study indicated that BC cells with E2F1 overexpression showed proliferation promotion and apoptosis inhibition, and NF-κB inhibitor treatment partially alleviated these effects. Therefore, E2F1 may promote BC progression through NF-κB. Collectively, the above results suggested that GSG2/p53/E2F1/NF-κB signaling pathway participated in the promotion of BC progress.

## Conclusion

In summary, GSG2 facilitated the development and progression of BC through MDM2-mediated ubiquitination of E2F1, which may be a promising candidate target with potential therapeutic value.

### Supplementary Information

**Additional file 1: Fig. S1. **Effective sequence screening targeting GSG2 and the effect of GSG2 knockdown on apoptosis-related protein expression. **Fig. S2. **Functional enrichment analysis and differential gene expression detection after GSG2 knockdown. **Fig. S3. **The knockdown of E2F1 and the overexpression and pathway enrichment of GSG2 in tumor cells were determined. **Table S1.** Primer sequence for PCR. **Table S2.** Antibodiesused in western blotting and Co-IP. **Table S3.** Cox multivariate analysis of GSG2expression in BC and other clinical features.

**Additional file 2.** Summary of differential expression gene after GSG2 knockdown.

## Data Availability

The datasets used and/or analysed during the current study are available from the corresponding author on reasonable request.
