# Supplementary material for: GSG2 facilitates the progression of human breast cancer through MDM2-mediated ubiquitination of E2F1
Source: J Transl Med. 2023 Aug 3;21:523. doi: 10.1186/s12967-023-04358-2 (PMC10398932; doi:10.1186/s12967-023-04358-2)
Supplement: Supplementary file 1 — Additional file 1: Fig. S1. Effective sequence screening targeting GSG2 and the effect of GSG2 knockdown on apoptosis-related protein expression. Fig. S2. Functional enrichment analysis and differential gene expression detection after GSG2 knockdown. Fig. S3. The knockdown of E2F1 and the overexpression and pathway enrichment of GSG2 in tumor cells were determined. Table S1. Primer sequence for PCR. Table S2. Antibodiesused in western blotting and Co-IP. Table S3. Cox multivariate analysis of GSG2expression in BC and other clinical features. [file 12967_2023_4358_MOESM1_ESM.docx]

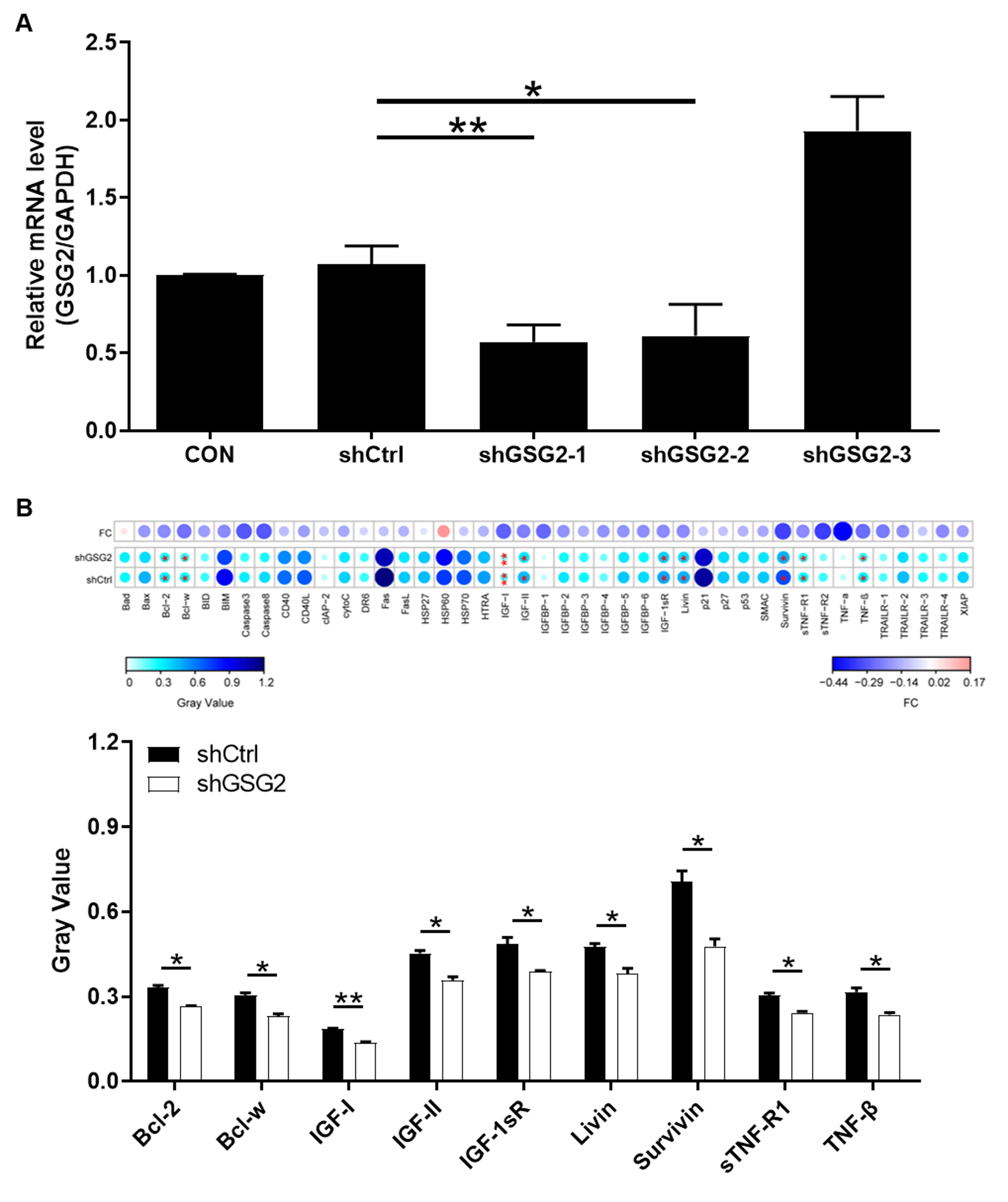


**Fig. S1** (A) The mRNA expression level of GSG2 was detected in 3 different shRNAs against GSG2 sequences. (B) The expression of 43 apoptosis-related regulators of MDA-MB-231 cells after knockdown of GSG2 was detected using apoptotic antibody array kit. The data were presented as the mean ± SD (n ≥ 3), *P<0.05, **P<0.01.


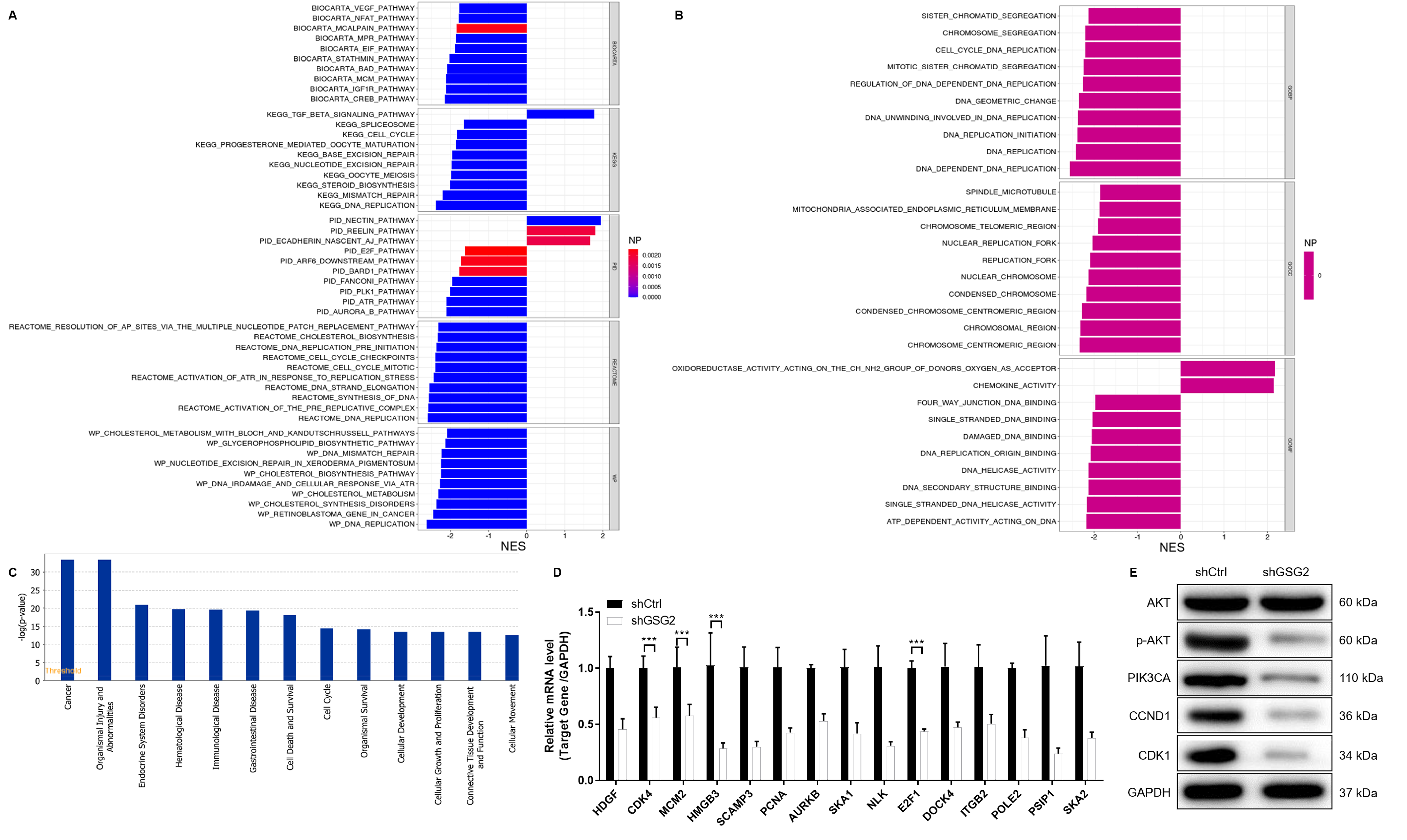


**Fig. S2** (A-B) Gene Set Enrichment Analysis (GSEA) was carried out based on shGSG2 vs shCtrl expression profile data. Ranked by significance P value, the top 10 gene sets that were most significantly concentrated in the classical pathways of KEGG, BIOCARTA, PID, REACTOME and WIKIPATHWAYS databases are shown. (C) The enrichment of the DEGs in IPA disease and function was analyzed by IPA. (D) The mRNA expression of most significant DEGs of MDA-MB-231 cells after knockdown of GSG2 was measured by qPCR. (E) The protein expression of AKT, PIK3CA, CCND1 and CDK1 in MDA-MB-231 cells after knockdown of GSG2 was measured by WB.


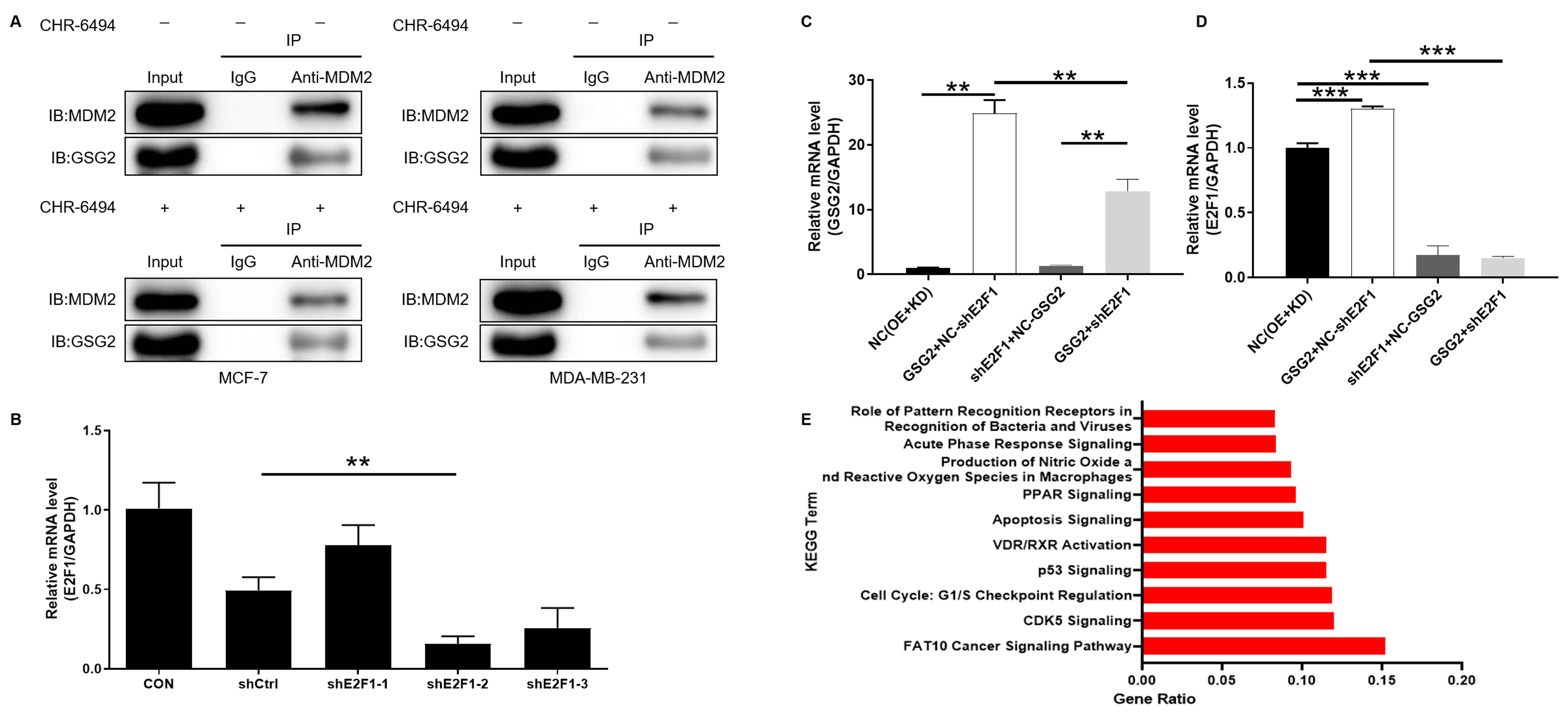


**Fig. S3** (A) The CO-IP was performed in MCF-7 and MDA-MB-231 cells treated with or without GSG2 kinase (CHR-6494). (B) The expression level of E2F1 was detected in 3 different shRNAs against E2F1 sequences. (C-D) The mRNA expression of GSG2/E2F1 in NC (OE+KD), GSG2+NC-shE2F1, shE2F1+ NC-GSG2 and GSG2+shE2F1 groups was evaluated qPCR. (E) The impact of GSG2 knockdown on the Kyoto Encyclopedia of Genes and Genomes (KEGG) was analyzed used the database for Annotation Visualization and Integrated Discovery (DAVID). The data were presented as the mean ± SD (n ≥ 3), *P<0.05, **P<0.01, ***P<0.001.

Table S1. Primer sequence for PCR.

| Gene | Upstream primer sequence | Downstream primer sequence | Amplified fragment size (bp) |
| --- | --- | --- | --- |
| GAPDH | TGACTTCAACAGCGACACCCA | CACCCTGTTGCTGTAGCCAAA | 121 |
| Haspin | GGAAGGGGTGTTTGGCGAAGT | TGAGGAGCAAGGGAGGGTAAG | 251 |
| IL6 | AAAGGCTGTGCTCTTGGTGA | TGGGACTCCTGGGAATACTG | 84 |
| CDK2 | ATCGCAAATGCTGCACTACG | GGTCACATCCTGGAAGAAAGG | 82 |
| HDGF | AGCCCAACAAGAGGAAAGGG | ACTGATAGCCGGAAGCCTTG | 81 |
| TGFBI | TGCTCCCACAAATGAAGCCT | GCCTCCGCTAACCAGGATTT | 127 |
| CDK4 | CTACCAGATGGCACTTACACCC | GCAAAGATACAGCCAACACTCC | 117 |
| MCM2 | CCAATGGCTTCCCTGTCTT | TCATCGGTCAGTTCCCCTAC | 91 |
| HMGB3 | ACCGTCTGGATTCTTCCTGTT | GATGTAAGGCTGCTTTTCACTG | 142 |
| KIF15 | CTCTCACAGTTGAATGTCCTTG | CTCCTTGTCAGCAGAATGAAG | 114 |
| SCAMP3 | GGCTTTGGGCTTTCTATCCT | GCCTTATACATGGGGCGGTA | 80 |
| PCNA | GGCGTGAACCTCACCAGTATGT | CGTTATCTTCGGCCCTTAGTGTA | 82 |
| AURKB | CATGGAGGAGTTGGCAGATG | CCTTGAGCCCTAAGAGCAGAT | 95 |
| SKA1 | TCCCATTTGCCTCAAGTAACAG | GGAGGCTTCTTTACGGGTTC | 107 |
| NLK | ACTCAGTATTATCGGGCTCCAG | CATCCCACAGACCAGATGTCA | 80 |
| E2F1 | CACTTTCGGCCCTTTTGCTC | GTGCTCTCACCGTCCTACAC | 128 |
| DOCK4 | CATTACTGCCCGGCTTGACT | GGATCCACCATTGCGTACTCTT | 81 |
| ITGB2 | TGACGCTTTACCTGCGACCA | CGCCACCTAGCTTCTTGACATT | 147 |
| CDCA7 | TCAGGGTGGCGATGAAGTT | GAATCGGAGTTGGAATCAGTCA | 109 |
| POLE2 | TGAGAAGCAACCCTTGTCATC | TCATCAACAGACTGACTGCATTC | 84 |
| PSIP1 | AGGGGTTACTTCAACCTCCG | CGTTTTCGATCTGCTGCTTC | 147 |
| SKA2 | AAAGTAGAGAGGAGGGGGCA | ATTGGAGACTGGGTAACGCC | 129 |

Table S2. Antibodies used in western blotting and Co-IP

| Primary antibodies | Dilution | Source species | Company | Catalog No. |  |
| --- | --- | --- | --- | --- | --- |
| Haspin | 1:1000 | Rabbit | Abcam | ab21686 |  |
| N-cadherin | 1:1000 | Rabbit | Abcam | ab18203 |  |
| Vimentin | 1:1000 | Rabbit | Abcam | ab92547 |  |
| Snail | 1:1000 | Rabbit | CST | 3879S |  |
| CDK4 | 1:1000 | Rabbit | Abcam | ab108357 |  |
| E2F1 | 1:1500 | Rabbit | Abcam | ab179445 |  |
| HMGB3 | 1:1000 | Rabbit | Abcam | ab75782 |  |
| KIF15 | 1:2000 | Rabbit | fine test | FNab04551 |  |
| Ubiquitin | 1:1000 | Mouse | Santa Cruz | sc-47721 |  |
| MDM2 | 1:2000 | Rabbit | Proteintech | 27883-1-AP |  |
| GAPDH | 1:3000 | Rabbit | Bioworld | AP0063 |  |
| Secondary antibody | Dilution |  | Company | Catalog No. |  |
| HRP Goat Anti-Rabbit IgG | 1:3000 |  | Beyotime | A0208 |  |
| Goat Anti-Mouse | 1:3000 |  | Beyotime | A0216 |  |

Table S3. Cox multivariate analysis of GSG2 expression in BC and other clinical

| Type | Hazard ratio | P |
| --- | --- | --- |
| GSG2 | 1.16 (1-1.36) | 0.057 |
| Sex | 2.16 (0.3-15.8) | 0.447 |
| Age | 1.04 (1.02-1.05) | 2.95E-07 |
| AJCC_stage | 1.82 (1.12-2.94) | 0.0149 |
| T_stage | 0.99 (0.74-1.33) | 0.949 |
| N_stage | 1.15 (0.87-1.53) | 0.33 |
| M_stage | 1.35 (0.66-2.75) | 0.411 |

features.
